# Protocol for a thematic synthesis to identify key themes and messages from a palliative care research network

**DOI:** 10.1186/s13104-016-2282-1

**Published:** 2016-10-21

**Authors:** Emma Nicholson, Tara Murphy, Philip Larkin, Charles Normand, Suzanne Guerin

**Affiliations:** 1 All Ireland Institute of Hospice and Palliative Care, 2nd Floor Education and Research Centre, Our Lady’s Hospice and Care Services, Harold’s Cross, Dublin, 6W Ireland; 2 UCD School of Nursing, Midwifery and Health Systems, UCD College of Health Sciences Belfield, Dublin, 4 Ireland; 3 Trinity College Dublin, School of Medicine, 3-4 Foster Place, Dublin, 2 Ireland; 4 UCD School of Psychology, John Henry Newman Building, University College Dublin, Belfield, Dublin, 4 Ireland

**Keywords:** Research networks, Ireland, Thematic synthesis, Dissemination

## Abstract

**Background:**

Research networks that facilitate collaborative research are increasing both regionally and globally and such collaborations contribute greatly to knowledge transfer particularly in health research. The Palliative Care Research Network is an Irish-based network that seeks to create opportunities and engender a collaborative environment to encourage innovative research that is relevant for policy and practice. The current review outlines a methodology to identify cross-cutting messages to identify how dissemination outputs can be optimized to ensure that key messages from this research reaches all knowledge users.

**Methods/design:**

Preferred reporting items for systematic review and meta-analysis protocol guidelines will inform the search and analysis plan to ensure that the synthesis of the data is as rigorous as possible. An approach based on critical interpretative synthesis will be adapted to include a thematic synthesis for the identification of higher-order themes and messages from a body of dissemination products generated by the Palliative Care Research Network.

**Discussion:**

The thematic synthesis outlined in the present protocol offers a novel method of synthesising data from a focused research network that employs a variety of dissemination materials as a means of identifying key themes and messages from a specific body of research. The high-level themes and messages will be identified from the thematic synthesis, widely disseminated and targeted towards a range of stakeholders and knowledge users such as carers, health and social care professionals, policy makers and researchers.

**Electronic supplementary material:**

The online version of this article (doi:10.1186/s13104-016-2282-1) contains supplementary material, which is available to authorized users.

## Background

Palliative care is defined as an approach that improves the quality of life of patients experiencing life-limiting or life-threatening conditions, as well as their families, through the prevention and relief of suffering [1]. Palliative care is unique in health settings as it often requires a different approach than that most often expected of health care professionals [2], where there is an increased focus on psychosocial and spiritual needs rather than purely medical needs [1]. In the Republic of Ireland alone, 80 % of deaths that occurred between 2007 and 2011 were due to chronic and life-limiting conditions traditionally associated with palliative care needs [3]. Across both the Republic of Ireland and Northern Ireland, the over-65 population is expected to increase by 169 % by 2041, with this group making up approximately 22–24 % of the population on the island [4]. These figures have considerable implications for service provision in palliative care on the island of Ireland given that advanced chronic conditions increase with older age. Indeed, over 65’s made up 82 % of palliative care needs in Ireland over a 5 year period [3] and the sharpest increase in deaths has occurred in those aged 85 years and older [5]. Thus, with an increased need for palliative care on the island of Ireland comes an increased need for research and networking that encourages engagement within a collaborative environment and supports the development of excellent, high quality, clinically-relevant and innovative research projects. The palliative approach is not exclusive to one particular discipline within the healthcare sector and often requires inter-disciplinary partnership across many healthcare providers within a healthcare setting [6]. Thus, palliative care research could adopt a similar approach whereby research teams and networks consist of individuals from a wide range of academic and clinical backgrounds.

Research as a team endeavour has risen dramatically in the past decades [7] and indeed, health research requires real-world collaboration between researchers and health care professionals [8]. In relation to palliative care, the European Association for Palliative Care Research Network (EAPC RN [9]) was founded in 1996 on the premise that networks are essential for palliative care research [10].

Research networks that facilitate collaborative research are increasing both regionally and globally and such collaborations contribute greatly to knowledge transfer [11], namely when collaborations between researchers and health care providers are encouraged [12]. For instance, the Cancer Experiences Collaborative (CECo) was a UK-based network that has highlighted the benefits of collaborative multi-disciplinary research networks such as engendering capacity building among members and encouraging user engagement [13].

One example of a formal structured research network is the All Ireland Institute for Hospice and Palliative Care (AIIHPC) Palliative Care Research Network (PCRN), which is an all-Ireland network that aims to respond to the growing need for research in palliative care. The AIIHPC PCRN was established in 2012 and is a collective of researchers committed to building research capacity, conducting high-quality collaborative research and extending knowledge to support better policy and practice in palliative care. The PCRN promotes collaborative research efforts through sustained programmes of research that aim to further enhance learning and exchange. Collaborative research is ideal in health care settings as it requires greater efforts towards knowledge translation which is crucial in order to foster successful implementation [14]. Given the iterative nature of knowledge transfer and learning [12] and the breadth of activity and evidence from the PCRN so far, a synthesis of evidence obtained from this research is timely. The principles of the PCRN incorporate public and service user engagement that engenders effective dissemination and the current protocol endeavours to reflect the operationalisation of these principles.

The current team has designed the present protocol with a view of looking outward to comparable reviews that aim to carry out an interpretive rather than integrative review of a specific body of literature. As stated above, synthesis is a key component of knowledge transfer particularly when looking to promote key messages from a body of literature [15] and thus, the present protocol aims to provide a synthesis of a specific source of research resulting in key themes and messages that will be used to target a wide range of knowledge users.

## Methods/design

### Aim

The purpose of this review is to conduct a thematic synthesis to identify high-level messages and themes from projects within the PCRN.

A broad research question will be adopted at the outset as the researchers will allow for the themes and messages to emerge organically as the review progresses, rather than setting a highly specific research question [16]. The main aim is the identification of key messages and themes from dissemination materials from across the PCRN. Critically, messages identified will be relevant to all knowledge users of the evidence produced by the PCRN (see supplementary materials for details of the projects in the PCRN).

### Study design

Preferred reporting items for systematic review and meta-analysis protocol (PRISMA P) guidelines [17] will inform the protocol for the current review with divergences from the guidelines implemented to meet the specific needs of the review. It is the culmination of a collaborative effort between University College Dublin (UCD), as an academic centre, and AIIHPC, as a partner organisation, which will facilitate access to multiple stakeholders. Collaborative research is key within palliative care to ensure the production of clinically-relevant research that is strategic and inter-disciplinary in its approach [12, 18].

Given the purpose of the review is to generate key themes and messages, the main focus of the review will be qualitative data, however, quantitative data may emerge during data extraction. Such data may include demographics and sample sizes from the studies and a tabulation of the frequency of products from the studies (e.g., number of dissemination products per studies), which will be included in the synthesis. To allow for synthesis of both qualitative and quantitative data, an approach similar to that taken by Dixon Woods et al. [16] will be adopted in the form of a critical interpretative synthesis (CIS). CIS draws heavily from traditional systematic review methodologies while incorporating recent developments with regards to interpretative synthesis [16]. Developed as a ‘mid-range’ theoretical account of existing evidence that aims to have wide explanatory scope and empirical applicability, it was derived from meta-ethnography [19] to include the use of quantitative data [16]. The current authors will draw from the methodology implemented by Dixon Woods et al. [16] whereby an organic process emerged which appropriated a systematic review protocol but allowed for adaptation based on the requirements of the review. For instance, a purposive sampling procedure utilised by Dixon Woods et al. [16] rather than the traditional inclusion criteria will be adapted for the current review given that the purpose of this review is to determine higher-order messages rather than produce an integrative review of research within the PCRN. Thematic synthesis will be implemented at the analysis stages to allow for the generation of the key messages and themes.

The following five steps will be carried out (1) setting the research question, (2) searching the literature; (3) sampling; (4) determination of quality; (5) data extraction; and (6) thematic synthesis.

### Search the literature

A purposive structured search of products from the PCRN will be carried out in an exhaustive manner to ensure that all relevant materials are collected. In the context of the current review, materials include conference presentations, study protocols, published peer-reviewed papers/abstracts, unpublished manuscripts, internal symposia, tweets/LinkedIn/ResearchGate/Facebook information, workshops/masterclasses, interim/final reports to AIIHPC and HRB, documents related to archived datasets, newsletters and feedback to participants. A traditional search of databases will be implemented to look for output from projects within the PCRN.

An infographic (see Additional file 1) was designed by the research team as a means to launch the project to the PCRN and wider palliative care community and invited researchers from each project in the PCRN (see Additional File 2) to send the authors their dissemination materials.

The most recent annual reports for the projects will be screened as a means to gather citations of all reported dissemination activities including published and in preparation peer-reviewed papers and abstracts. Any posters or oral presentations presented at national and international conferences, workshops or seminars that are referred to in the annual report will be recorded and sought from the researchers.

Traditional online databases and other online sources will be used to search for materials which are relevant to the projects within the PCRN and that are available through such means. The names of all researchers within the PCRN will be used as the search terms using ScienceDirect, PsycInfo, CINAHL and PubMed.

The researchers in the PCRN will also be contacted to provide unpublished manuscripts, abstracts, and theses (where applicable) that arose from the HRB funded and aligned projects. The Twitter pages, ResearchGate and LinkedIn profiles of all the researchers within the PCRN will be collected and recorded and their accounts searched for any discussion or comments related to the PCRN projects.

A database of all dissemination materials and products will be created once all items have been gathered. Materials collected during sampling will be recorded using a Microsoft Excel database.

### Sampling

No eligibility criteria for the studies will be applied since all data production is considered relevant. Given the exclusive nature of the search to projects within the PCRN, quality appraisal techniques will be applied to the materials to ensure rigor in the selection of materials and products collected in the search stage.

### Determination of quality

**Fig. 1:**
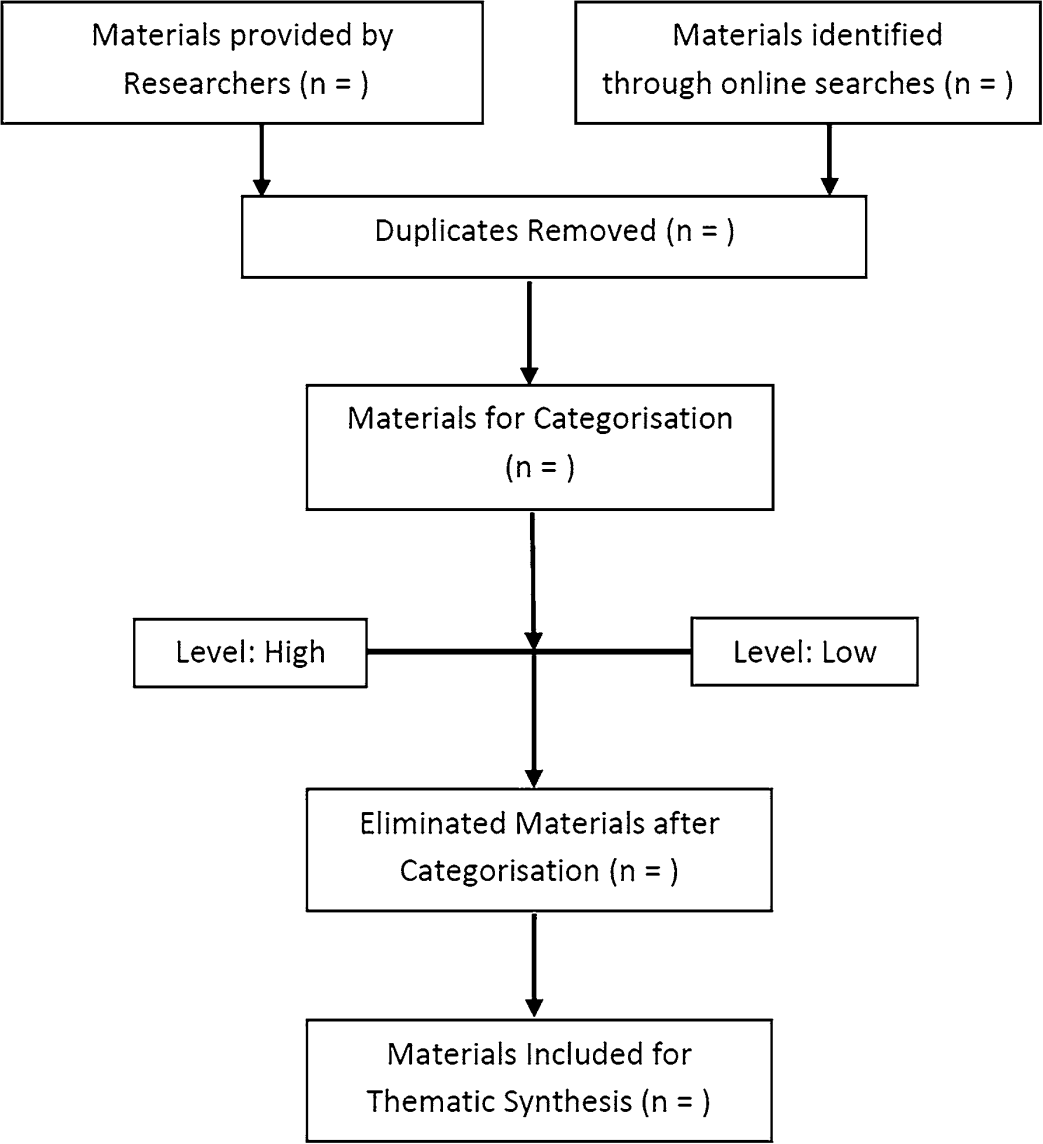
Flow chart for the current review

Products will be categorised on two levels (e.g., high or low) with greater weight [20] assigned to those materials identified as high. The features of this categorisation will include for instance the level of detail of content contained in the product in relation to palliative care or the type of content included in the materials (e.g., results of a study or announcement of a presentation). Decisions regarding categorisation will be contingent on the content of the materials collected and will be made as the appraisal process is on-going and will be discussed by the authors throughout the review process. Materials may be eliminated from the review during this stage if they are deemed to not be relevant to the review. See Fig. 1 for a description of the method.

### Data extraction

A data extraction protocol will be implemented to extract the relevant data from materials collected (see Table 1).

**Table 1:** Data extraction form

1	The title of the project with which the dissemination product is associated
2	The author(s) of the product
3	Designated category of the product (high or low)
4	Target audience (e.g., where was it presented, focus of the journal)
5	Method (sample, location, ??)
6	Outcomes from the research stated in the product(s)

Partial double extraction will be implemented to reduce error rate during this process [21]. Two reviewers will review the selected materials and extract data and a third reviewer will be consulted where discrepancies occur.

### Thematic synthesis

The data that emerge from data extraction will be subjected to thematic synthesis [22]. Similar to analysis of primary qualitative datasets, thematic synthesis involves the systematic coding of data and generating of descriptive and analytical themes. It is an inductive approach which is critical given the aim to generate higher-order themes and key messages from the projects in the PCRN. It is a three stage process [22] which begins with line-by-line coding of text where findings from the materials collected will be entered word-for-word into the database and each line of text will be coded according to its meaning and content. Following this step is the development of descriptive themes which involves translating the concepts from one study to another and a hierarchical structure will be created by grouping the codes based on similarities and differences between the codes. Finally, the generation of analytical themes that go beyond the content of the original articles is a critical stage where descriptive themes are used to determine the key messages. Themes and messages will be reviewed independently to consider implications and then discussed as a group to allow for the emergence of more abstract messages and themes that go beyond the content in the original materials.

## Discussion

The current review offers a method of synthesising data from a focused research network that employs a variety of dissemination materials as a means of identifying key themes and messages from a specific body of research (i.e. the PCRN). Given the significant role that research networks play in palliative care research [10] and the importance of knowledge exchange and dissemination in the implementation of health research [12], the review represents an endeavour to maximise the learning and impact of a research network by incorporating all forms of dissemination activity available. For example, users/carers, health and social care professionals will take part in reflection groups to allow for further interpretation of the themes and messages identified.

### Dissemination plan

One means for researchers to effectively target specific knowledge users is with clear and concise messages aimed at a specific audience delivered in a way that the recipients want and that are supported by a credible body [23]. The results of the current review will be targeted at a range of stakeholders and knowledge users such as researchers, health and social care professionals as well as users/family carers. A dissemination plan based upon the model developed by [24] will be implemented and involve messages and themes identified transmitted in a series of short videos, podcasts, policy briefs and newsletters with specialist input from key stakeholders such as researchers, practitioners, policy makers and users and carers of palliative care services.

## Additional files

**Additional file 1.** KINDLE Project Infographic.

**Additional file 2.** Details of projects from the Palliative Care Research Network that will contribute to the thematic synthesis.

## Abbreviations

AIIHPC: All Ireland Institute of Hospice and Palliative Care
PCRN: Palliative Care Research Network
CIS: critical interpretative synthesis
PRISMA P: preferred reporting items for systematic review and meta-analysis protocol
EAPC RN: European Association for Palliative Care Research Network
CECo: Cancer Experiences Collaborative

## References

[1] World Health Organisation. WHO Definition of Palliative Care. http://www.who.int/cancer/palliative/definition/en/. Accessed 28 Mar 2016.

[2] Morrison RS, Meier DE (2004). Palliative care. N Engl J Med.

[3] Kane PM, Daveson BA, Ryan K, McQuillan R, Higginson IJ, Murtagh FEM (2015). The need for palliative care in ireland: a population-based estimate of palliative care using routine mortality data, inclusive of nonmalignant conditions. J Pain Symptom Manage.

[4] McGill P. Illustrating ageing in ireland north and south key facts and figures. 2010. http://www.cardi.ie/publications/illustratingageinginirelandnorthsouthkeyfactsandfigures. Accessed on 13 Apr 2016.

[5] StatBank: Death occurring by area of residence, age at death, year and sex. Central Statistics Office. Cork, Ireland. Accessed on 25 May 2016.

[6] Sawatzky R, Porterfield P, Lee J, Dixon D, Lounsbury K, Pesut B (2016). Conceptual foundations of a palliative approach: a knowledge synthesis. BMC Palliat Care..

[7] Collaboration Whitfield J (2008). Group theory. Nature.

[8] Girot E (2008). Developing researchers. Nurse Manage..

[9] European Association for Palliative Care. About the EAPC Research Network. http://www.eapcnet.eu/Themes/Research/AbouttheEAPCResearchNetwork.aspx. Accessed on 9 May 2016.

[10] Hanks G (2008). The EAPC research network, the EPCRC and the sunshine in the Lofoten islands. Palliat Med.

[11] Adams J (2012). The rise of research networks. Nature.

[12] Canadian Institute of Health Research. 2009. http://www.cihr-irsc.gc.ca/e/29418.html. Accessed on 14 Mar 2016.

[13] Payne S, Seymour S, Moliassiotis A, Froggatt K, Grande G, Lloyd-Williams M (2011). Benefits and challenges of collaborative research: lessons from supportive and palliative care. BMJ Support Palliat Care..

[14] Bennet MI, Davies EA, Higginson IJ (2010). Delivering research in end-of-life care: problems, pitfalls and future priorities. Palliat Med..

[15] Gagliardi AR, Legare F, Brouwers MC, Webster F, Wiljer D, Badley E (2011). Protocol: developing a conceptual framework of patient mediated knowledge translation, systematic review using a realist approach. Implement Sci..

[16] Dixon Woods M, Cavers D, Agarwal S, Annandale E, Arthur A, Harvey J (2006). Conducting a critical interpretative synthesis of the literature on access to healthcare by vulnerable groups. BMC Med Res Methodol.

[17] Moher D, Clarke M, Ghersi D, Liberati A, Petticrew M, Shekelle P, Stewart LA, PRISMA P Group (2015). Preferred reporting items for systematic review and meta-analysis protocols (PRISMA-P) 2015: elaboration and explanation. BMJ.

[18] McIlfatrick SJ, Murphy T (2013). Palliative care research on the island of Ireland over the last decade: a systematic review and thematic analysis of peer reviewed publications. BMC Palliat Care..

[19] Noblit G, Hare R (1988). Meta-ethnography: synthesising qualitative studies.

[20] Rutter D, Francis J, Coren E, Fisher M. SCIE systematic research reviews: guidelines (2^nd^ Edition). Social Care Institute for Excellence. 2010. http://www.scie.org.uk/publications/researchresources/rr01.asp Accessed 11 May 2016.

[21] Buscemi N, Hartling L, Vandermee B, Tjosvold L, Klassen TP (2006). Single data extraction generated more errors than double data extraction in systematic reviews. J Clin Epidemiol.

[22] Thomas J, Harden A (2008). Methods for the thematic synthesis of qualitative research in systematic reviews. BMC Med Res Methodol.

[23] Gagnon ML (2011). Moving knowledge to action through dissemination and exchange. J Clin Epidemiol.

[24] Prihodova L, Guerin S, Kernohan WG (2015). Knowledge transfer and exchange frameworks in health and their applicability to palliative care: scoping review protocol. J Adv Nurs.

