# Supplementary material for: Protocol for a thematic synthesis to identify key themes and messages from a palliative care research network
Source: BMC Res Notes. 2016 Oct 21;9:478. doi: 10.1186/s13104-016-2282-1 (PMC5073737; doi:10.1186/s13104-016-2282-1)
Supplement: Supplementary file 1 — Additional file 1. KINDLE Project Infographic. [file 13104_2016_2282_MOESM1_ESM.pdf]

AIIHPC Palliative Care Research Network

KINDLE Project

Knowledge Innovation
Dissemination
Learning Exchange

Project Background: In 2015, the Health Research Board (HRB) awarded AIIHPC funding under the knowledge exchange and dissemination scheme (KEDS) as a means to enhance knowledge transfer activities and increase the impact of the projects in the Palliative Care Research Network (PCRN). This project was initiated in December 2015.

Project team includes Dr Emma Nicholson AIIHPC (Project Manager), Dr Tara Murphy AIIHPC and Dr Suzanne Guerin UCD/AIIHPC (Project Co-Leads).

1 Partners

(Who is this relevant to?)

Unlocking **Knowledge and Innovation** contained within the PCRN and aligned projects including HRB funded projects (Social Justice and Measurement & Evaluation Strands), Knowledge Transfer and Exchange (KTE) Project, and Clinical, Doctoral & Postdoctoral Fellowships

2 Products

(What are we seeking?)

Dissemination 'products' including conference presentations, study protocols, published peer-reviewed papers/abstracts, unpublished manuscripts, internal symposia, tweets/LinkedIn/ResearchGate/Facebook information, workshops/masterclasses, interim/final reports to AIIHPC and HRB, documents related to archived datasets, newsletters, feedback to participants.

3 Process

(What will we do with the dissemination products?)

A rigorous and systematic synthesis of data to identify cross-cutting and high-level messages and themes from across the projects in the PCRN.

4 Output

(What will happen to these key messages/ themes?)

Short videos, podcasts, policy briefs and newsletters with specialist input from key stakeholders such as researchers, practitioners, policy makers and users and carers of palliative care services. The aim is to further enhance **learning and exchange** activities of the research to ensure that critical themes and messages from the PCRN intersect all knowledge users.

AIIHPC
Palliative Care Research Network

Health Research Board

UCD DUBLIN

A structured search will be undertaken to identify dissemination products available through traditional online sources and databases.

Further products should be sent to
Dr Emma Nicholson in AIIHPC.
Email: enicholson@aiihpc.org

www.aiihpc.org **@AIIHPC** **#KINDLEProject**
