# Supplementary material for: Protocol for a thematic synthesis to identify key themes and messages from a palliative care research network
Source: BMC Res Notes. 2016 Oct 21;9:478. doi: 10.1186/s13104-016-2282-1 (PMC5073737; doi:10.1186/s13104-016-2282-1)
Supplement: Supplementary file 2 — Additional file 2. Details of projects from the Palliative Care Research Network that will contribute to the thematic synthesis. [file 13104_2016_2282_MOESM2_ESM.docx]

**Additional File 2.** Details of projects from the Palliative Care Research Network that will contribute to the thematic synthesis

| **Project Title** | **Research Team** | **Host Institution(s)** | **Funding Body** |
| --- | --- | --- | --- |
| Identifying and addressing the needs of people with serious mental Illness | Ann Sheridan^1^, Gerard Leavey^2^, Sarah Walsh^1^ | ^1^University College Dublin (UCD)/ ^2^University of Ulster (UU) | HRB |
| Identifying and Addressing the Needs of People with Intellectual Disability | Mary McCarron^1^, Karen Ryan^2,3^, Janet O’Farrell^1^ | ^1^Trinity College Dublin (TCD)/ ^2^St Francis’ Hospice (SFH)/^3^UCD | HRB |
| An Exploration of access, decision making and experiences of palliative care services for families of children with a non-malignant life-limiting condition | Gemma Kiernan^1^, Honor Nichol^2^, Fiona Hurley^1^ | ^1^Dublin City University (DCU)/ ^2^TCD | HRB |
| Exploring Dimensions of Inequality in Current Palliative Care Provision for Carers of People with Advanced Heart Failure in Ireland | Donna Fitzsimmons, Sonja McIlfatrick, Leanne Doherty | UU | HRB |
| Towards Improved diagnosis and symptom management in palliative care | David Meagher^1^, Karen Ryan^2, 3^,  Mas Mohammed^1^, Brid Davis^1^ | ^1^University of Limerick (UL)/ ^2^SFH/^3^UCD | HRB |
| Development & evaluation of a psycho-educational intervention for patients with refractory cachexia & their lay carers | Sam Porter, Joanne Reid, David Scott | Queen’s University Belfast (QUB) | HRB |
| Eliciting preferences for complex packages of palliative care – extension of IARE | Charles Normand, Bridget Johnson | TCD | HRB |
| Developing & implementing a ‘System’ of structured network-wide dissemination & knowledge transfer activities | W. George Kernohan^1^, Suzanne Guerin^2^, Lucia Prihodova^2^, Mary Jane Brown^1^ | ^1^UU/ ^2^UCD | AIIHPC |
| General practitioners’ perceptions on palliative care for individuals with Alzheimer’s disease and other progressive dementias | Kevin Brazil^1^, Karen Galway^1^, Jenny van der Steen^2^, Max Watson^3^ | ^1^QUB/^2^VU University Medical Centre Amsterdam/^3^Northern Ireland Hospice | HSC Research & Development Division: HSC Public Health Agency & The Atlantic Philanthropies |
| Promoting informed decision-making and effective communication through advance care planning for people living with dementia and their family carers | Kevin Brazil^1^, Mike Clarke^1^, Katherine Froggatt^1^, Peter Hudson^1^, George Kernohan^2^, Dorry McLaughlin^1^, Peter Passmore^1^ | ^1^QUB/^2^UU | Care to Know Centre, Ontario, Canada |
| Consensus project on quality in palliative care day services | Noleen McCorry^1^ , Martin Dempster^2^ | ^1^Marie Curie Hospice Belfast/^2^QUB | Marie Curie UK |
| Costs and effectiveness of UK palliative care day services: a three-centre mixed methods study of impact upon patients and family carers | George Kernohan, Joanne Jordan | UU | Marie Curie UK |
| Pain assessment and management for patients with advanced dementia nearing the end of life | Carole Parsons^1^, Bannin De Witt Jansen^1^, Kevin Brazil^1^, Peter Passmore^1^, Hilary Buchanan^2^, Doreen Maxwell^3^, Sonja McIlfatrick^4^, Sharon Morgan^5^, Max Watson^6^ | ^1^QUB/ ^2^Patient and Public Involvement Representative/ ^3^Kerrsland Surgery GP Practice/^4^UU/^5^Marie Curie Hospice/^6^Northern Ireland Hospice | HSC Research & Development Division: HSC Public Health Agency & The Atlantic Philanthropies |
| Delayed intervention randomised controlled trial to assess the effectiveness of a new model of social and practical support for community dwelling adults living with advanced life-limiting illness in Limerick, Ireland | Kathy McLoughlin | Maynooth University | AIIHPC/Irish Cancer Society |
| Social Justice and the conceptualisation of the body-self in palliative care in Ireland | Kathleen Lynch, Luciana Lolich | UCD | HRB |
| Developing a cancer cachexia rehabilitation intervention for people with inoperable advanced non-small cell lung cancer | Cathy Payne | Public Health Agency | AIIHPC |
| Transition to adult services by young people with life-limiting conditions in Belfast and Dublin: a realist evaluation using mixed methods (TASYL study) | Helen Kerr | QUB | AIIHPC |
| ­­­­­­­­­­­­­­­­­­­­­­­­What are the clinical effectiveness and cost-effectiveness of different organizational models of community specialist palliative care provision? | Charles Normand^1^, Karen Ryan^2,3^, Sonja McIlfatrick^4^ | ^1^TCD/ ^2^SFH/ ^3^UCD/ ^4^UU | HSE, HRB |
